# Characterization of wheat *MYB* genes responsive to high temperatures

**DOI:** 10.1186/s12870-017-1158-4

**Published:** 2017-11-21

**Authors:** Yue Zhao, Xuejun Tian, Fei Wang, Liyuan Zhang, Mingming Xin, Zhaorong Hu, Yingyin Yao, Zhongfu Ni, Qixin Sun, Huiru Peng

**Affiliations:** State Key Laboratory for Agrobiotechnology and Key Laboratory of Crop Heterosis and Utilization (MOE), Beijing Key Laboratory of Crop Genetic Improvement, Department of Plant Genetics and Breeding, China Agricultural University, Beijing, 100193 People’s Republic of China

**Keywords:** Abiotic stress, Heat stress, MYB, Transgenic *Arabidopsis*, Wheat

## Abstract

**Background:**

Heat stress is one of the most crucial environmental factors, which reduces crop yield worldwide. In plants, the MYB family is one of the largest families of transcription factors (TFs). Although some wheat stress-related MYB TFs have been characterized, their involvement in response to high-temperature stress has not been properly studied.

**Results:**

Six novel heat-induced *MYB* genes were identified by comparison with previously established de novo transcriptome sequencing data obtained from wheat plants subjected to heat treatment; genomic and complete coding sequences of these genes were isolated. All six TaMYBs were localized in the nucleus of wheat protoplasts. Transactivation assays in yeast revealed that all six proteins acted as transcriptional activators, and the activation domains were attributed to the C-termini of the six wheat MYB proteins. Phylogenetic analysis of the six TaMYBs and R2R3-MYBs from *Arabidopsis* revealed that all six proteins were in clades that contained stress-related MYB TFs. The expression profiles of *TaMYB* genes were different in wheat tissues and in response to various abiotic stresses and exogenous abscisic acid treatment. In transgenic *Arabidopsis* plants carrying *TaMYB80* driven by the *CaMV 35S* promoter, tolerance to heat and drought stresses increased, which could be attributed to the increased levels of cellular abscisic acid.

**Conclusions:**

We identified six heat-induced *MYB* genes in wheat. We performed comprehensive analyses of the cloned *MYB* genes and their gene products, including gene structures, subcellular localization, transcriptional activation, phylogenetic relationships, and expression patterns in different wheat tissues and under various abiotic stresses. In particular, we showed that *TaMYB80* conferred heat and drought tolerance in transgenic *Arabidopsis*. These results contribute to our understanding of the functions of heat-induced *MYB* genes and provide the basis for selecting the best candidates for in-depth functional studies of heat-responsive *MYB* genes in wheat.

**Electronic supplementary material:**

The online version of this article (10.1186/s12870-017-1158-4) contains supplementary material, which is available to authorized users.

## Background

Heat stress is one of the most severe environmental factors restricting crop distribution and production [1, 2]. To overcome heat stress, plants adopt various strategies involving the perception of ambient temperature and the regulation of signaling networks [3, 4]. Gene regulation at the transcriptional level is one of the primary control points in biological processes in which transcription factors (TFs) play a key role by activating and/or repressing target genes. In vascular plant genomes, approximately 7% of the coding capacity is attributes to TFs [5], which implies complex gene regulation at the transcriptional level. Previous studies have revealed that several TF families, such as MYB, HSF, DREB, NAC, and bZIP, play important roles in response to stresses in plants [6–9]. For example, many of these TF genes were significantly induced by heat treatment in rice and wheat, as revealed by transcriptome analyses [10, 11].

MYB TFs are found in all eukaryotes. Based on the number of repeats of the MYB domain, the MYB TF family is divided into four major classes: 1R–MYB or MYB-related proteins (usually with a single repeat), R2R3-MYB (two repeats), 3R–MYB (three repeats), and 4R–MYB (four repeats). In plants, the MYB family is one of the largest families of TFs. So far, 204 MYB TFs have been identified in *Arabidopsis*, whereas there are 218 and 244 MYBs in rice (*Oryza sativa*) [12] and soybean (*Glycine max*) [13], respectively. Accumulated evidence has indicated that MYBs mediate stress-signaling pathways. *AtMYB41*, *AtMYB74*, *AtMYB102*, and *AtMYB108* play critical roles in drought and salt tolerance [14]. For example, *AtMYB41* transcripts are induced by drought and high salinity in *Arabidopsis*; and overexpression of *AtMYB41* in transgenic *Arabidopsis* increases plant tolerance to drought and high salinity and the expression of several stress-related genes [15, 16]. Another type of MYB TF, *AtMYB15*, participates in the ABA signaling pathway by targeting ABA biosynthesis (*ABA1*, *ABA2*), signaling (*ABI3*), and responsive genes (*AtADH1*, *RD22*, *RD29B*, *AtEM6*) [17]. In rice, the expression of *OsMYB2* is induced by cold, drought, and salt stresses. Analyses of transgenic rice plants overexpressing or repressing *OsMYB2* showed that *OsMYB2* increases tolerance to low temperature, salt, and drought treatments [18]. The rice *OsMYB48–1* gene plays a positive role in stress tolerance, and overexpression of *OsMYB48–1* provides salt and drought tolerance for transgenic rice plants [19]. *GbMYB5* was isolated from cotton, and the role of *GbMYB5* in response to drought stress has been recently characterized in cotton by VIGS and in tobacco by overexpression [20]. Silencing of *GbMYB5* in cotton decreased proline content and antioxidant enzyme activities and compromised the tolerance of cotton plantlets to drought stress, whereas overexpression of *GbMYB5* in tobacco increased tolerance to drought stress by decreasing water loss and elevating proline content and antioxidant enzyme activities. In wheat, several *MYB* genes involved in the response to multiple abiotic stresses have been identified, and transgenic *Arabidopsis* plants overexpressing *TaMYB2A*, *TaMYB19*, *TaMYB30-B*, or *TaMYB33* show increased tolerance to multiple abiotic stresses compared with wild-type (WT) plants [21–24].

In comparison to the rapid progress made in studies on the role of MYBs in response to drought and high salinity stress, the functions of MYBs in response to heat stress are not as well known. So far, only *AtMYB68, LeAN2*, and *OsMYB55* have been proposed to play a role in heat tolerance. In *Arabidopsis*, vegetative growth of *Atmyb68* mutants was significantly inhibited under high temperature compared with WT plants [25]. The overexpression of *LeAN2* in tomato caused anthocyanin accumulation and conferred increased tolerance to heat stress by maintaining a low level of reactive oxygen species and high non-enzymatic antioxidant activity [26]. In rice, overexpression analysis demonstrated that *OsMYB55* improved high temperature tolerance in transgenic rice plants by increasing expression of the downstream genes *OsGS1;2*, *GAT1*, and *GAD3*, which are involved in amino acid metabolism [27]. Recently, the constitutive expression of rice *OsMYB55* in maize induced several stress-related genes and resulted in improved plant growth and performance under high temperature and drought conditions [28]. Before the present study, wheat MYB genes involved in heat tolerance had not been identified.

Bread wheat (*Triticum aestivum* L.) is one of the most widely cultivated and important food and feed crops globally. High temperature affects the growth and productivity of wheat and reduces yields worldwide. To investigate the role of wheat *MYB* genes in plant tolerance to heat, we identified six heat-responsive *MYB* genes based on our previously published transcriptome data [10]. Further, we demonstrated that all six MYB TFs were transcriptional activators and we localized the positions of their activation domains (ADs) and we determined their subcellular locations. The expression levels of all six *TaMYBs* in different wheat tissues and under different stresses were analyzed by reverse transcription quantitative real-time PCR (RT-qPCR), and we also showed that the tolerance of transgenic *Arabidopsis* to heat and drought increased when *TaMYB80* was constitutively overexpressed. This study provides a useful reference for the selection of heat-responsive *MYB* genes in wheat for further functional analyses of these genes and their products.

## Methods

### Plant material and stress treatments

Heat and drought tolerant wheat cv. TAM107, which was released by Texas A&M University in 1984, was used for this study. For stress treatment experiments, seeds were sterilized in a solution of 1% NaClO, and plants were grown hydroponically in a light chamber with 16/8-h photoperiod (photosynthetic photon flux density or PPFD of 100 μmol m^−2^ s^−1^) at 22 °C/18 °C (day/night)and 70% relative humidity. Seven-day-old wheat seedlings were treated by submerging the roots in 1/2 Hoagland’s solution with 200 μM ABA, 200 mM NaCl, or 20% PEG 6000 (polyethylene glycol 6000). After treatment, plants were grown under constant (24 h per day) illumination at 22 °C and 70% relative humidity. For heat stress, the seedlings were transferred to another growth chamber at a constant temperature of 40 °C and with the same settings of light and humidity as the previous chamber. For each treatment, five seedlings were separately sampled at 0, 0.5, 1, 2, 4, 6, 12, and 24 h after treatment. For analyses of tissue-specific expression patterns of *TaMYBs*, roots, stems, flag leaves, 10–20 mm developing spikes, and spikes at flowering were collected from wheat plants grown under the same field conditions. Root samples were quickly rinsed with running tap water to remove soil, then rinsed with deionized water and dried with absorbent paper. All collected samples were quickly placed into liquid nitrogen and stored at −80 °C for RNA extraction.

### Extraction of genomic DNA and total RNA and cDNA synthesis

For cloning the genomic sequences of *TaMYBs*, genomic DNA was isolated from TAM107 young leaves using the CTAB method. To obtain the coding sequences of *TaMYBs* and to assess the expression patterns of cloned *TaMYBs*, total RNA was isolated from different samples using Trizol reagent according to the manufacturer’s protocol (TIANGEN, China) and treated with RNase-free DNase I (TaKaRa, Japan) to remove genomic DNA contamination. Two micrograms of total RNA were reverse transcribed using M-MLV reverse transcriptase (TaKaRa, Japan) following the manufacturer’s instructions.

### Gene cloning and sequence analysis

Candidate *MYB* gene sequences were selected from the transcriptome data of the wheat cultivar TAM107 generated in our laboratory [10]. According to the obtained sequences, specific primers for the six *TaMYBs* were designed using DNAMAN software (www.lynnon.com). DNA and cDNA were used as templates to amplify the DNA sequences and cDNA sequences of wheat MYBs, respectively. PCR was performed using high-fidelity PrimeSTAR Polymerase (TaKaRa, Japan) under the following conditions: 98 °C for 3 min; 34 cycles of 98 °C for 15 s, 58 °C for 30 s, and 72 °C for 90 s; followed by an extension step of 72 °C for 10 min. The PCR amplification products were ligated into the pEASY-blunt Cloning Vector (TransGen, China), and the resulting ligation mixtures were transformed into *E. coli* Trans1-T1 chemically competent cells (TransGen, China). The positive clones verified by PCR were sequenced.

Protein domains of TaMYBs were predicted using the SMART tool (http://smart.embl-heidelberg.de/), and their gene structures were obtained using the GSDS tool (http://gsds.cbi.pku.edu.cn/). Theoretical molecular weight and isoelectronic point (*p*I) were calculated using the ProtParam tool (http://web.expasy.org/protparam/). For phylogenetic analysis, 126 *Arabidopsis* R2R3-MYB protein amino acid sequences were downloaded from the NCBI website (http://www.ncbi.nlm.nih.gov/). The MYB protein amino acid sequences were aligned with the Clustal X version 2.0 program [29], and the phylogenetic tree was constructed using MEGA6 software with the neighbor-joining (NJ) method [30].

### Gene expression analysis

*TaMYB80* gene expression in transgenic *Arabidopsis* was analyzed by semi-quantitative reverse transcript (RT)-PCR. *Actin2* was used as the internal control. Tissue-specific gene expression patterns of *TaMYBs* and gene expression patterns of *TaMYBs* under various stress treatments were determined by RT-qPCR analysis. The RT-qPCR was conducted using an SYBR® Green reaction kit (TaKaRa, Japan) and a Bio-Rad CFX96 real-time system (http://www.bio-rad.com/) with gene-specific primers. The relative expression was calculated using the 2^-ΔΔCT^ method [31]. The wheat *β-actin* gene was used for standardization of target gene expression. The conditions for semi-quantitative RT-PCR and RT-qPCR have been described previously [32]. All experiments were performed with three biological replicates and three technical replicates. The primers are listed in Additional file 1: Table S1.

### Subcellular localization analysis

The coding regions of the six *TaMYBs* without the stop codon were amplified using gene-specific primers and fused to the N-terminal of the *green fluorescent protein* (*GFP*) gene sequence under the control of the *Cauliflower mosaic virus (CaMV) 35S* promoter. The subcellular localization of each TaMYB was assessed with transient expression after PEG-mediated transfection in wheat mesophyll protoplasts [33, 34] and was monitored 18 h after PEG-calcium transfection using a confocal microscope (FV1000, Olympus).

### Transcriptional activation assays in yeast

The transactivation experiment was conducted according to the manual of Yeast Protocols Handbook (Clontech). The full-length and truncated coding sequences of *TaMYBs* obtained by PCR amplification were fused in-frame to the pGBKT7 vector (Invitrogen), which contained the coding sequence of the GAL4 DNA-binding domain (BD) and *Trp* reporter gene. *Eco R*I restriction sites were introduced into the forward and the reverse gene-specific primers (Additional file 1: Table S1). The constructs, positive control pGBKT7–53 and negative control pGBKT7 were respectively transformed into the *Saccharomyces cerevisiae* AH109 strain (harboring the *HIS3* reporter gene) following the manufacturer’s recommended procedures (Clontech). After incubation at 28 °C for two days, positive clones were identified by PCR and plated onto synthetic defined (SD)/−Trp and SD/−Trp/−His media. The AH109 strain could not grow on the SD medium lacking Trp unless a functional *TRP1* gene was introduced, and the strain could not grow on the SD/−His medium without activation of a *HIS3* gene. Therefore, the growth of yeast cells on SD/−Trp medium showed that the pGBKT7 constructs were successfully transformed into yeast, and the growth of yeast on SD/−Trp/−His medium demonstrated the ability of the MYB proteins to activate the transcription of a reporter gene.

### *Arabidopsis* transformation and stress treatment

The coding sequence of *TaMYB80* was amplified from a plasmid by PCR using gene-specific primers containing attB sites. The PCR product was cloned downstream of the *CaMV 35S* promoter in the plant expression vector pB2GW7 using the Gateway cloning method. The construct was confirmed by sequencing. *Arabidopsis* (Col-0) transformation by the recombinant plasmid was performed via *Agrobacterium tumefaciens*-mediated floral-dip method [35]. Five-day-old transgenic seedlings were screened by spraying 0.1% (*v*/v) Basta, and the putative Basta-resistant plants were confirmed by RT-PCR. Homozygous T3 seeds of transgenic lines were used for phenotypic analyses.

For the germination assay under heat stress treatment, seeds of WT and transgenic lines were sterilized and subjected to 50 °C heat stress treatment for 1 h by submersion of the corresponding microtubes with seeds into a temperature-controlled water bath and then germinated on 1% agar plates. These plates were incubated at ambient temperature (22 °C) for 7 d before photographs were taken and the percentage of germinated seeds was calculated. Seeds were considered germinated when radicles had completely emerged from the seed coat. At least 50 seeds were assessed for each of five independent replicates. For the survival assay, 5-day-old seedlings were heat-treated at 45 °C for 1 h before being returned to 22 °C to grow for another two days after which photographs were taken and the survival rates were calculated. More than 40 plants of each line were used for analysis. For drought sensitivity assays, surface-sterilized WT and transgenic seeds were planted on a Murashige and Skoog medium and placed in a light chamber (22 °C, 16 h photoperiod). Seven-day-old seedlings were transferred to pots with mixed soil (rich soil:vermiculite = 2:1, *v*/v). Each pot contained four seedlings, which were grown under the same conditions for 21 days with sufficient watering before the application of drought. After two weeks of drought stress treatment by withholding water, the plants were re-watered and allowed to recover from the drought stress conditions.

### Determination of the water loss rate in *Arabidopsis*

To measure water loss, rosette leaves from three-week-old transgenic and WT plants were detached and weighed immediately. The samples were placed on a laboratory bench (humidity 40–50%, 22–24 °C) and weighed at designated time points. The water loss rate was calculated based on the initial fresh weight of samples. Five plants of each transgenic or WT line were used for this measurement, and all tests were repeated three times.

### Quantification of ABA levels in *Arabidopsis*

The ABA content was measured in rosette leaves of three-week-old transgenic and WT plants, with three biological replicates. For each replicate, at least 200 mg of *Arabidopsis* leaves were homogenized in liquid nitrogen, and 50 mg of the homogenized fresh weight of *Arabidopsis* leaves were used to measure ABA. ABA extraction and determination were performed as previously described [36].

### Statistical analyses

Differences between each transgenic line and WT were analyzed using Student’s *t*-test in Excel (Microsoft Office 2016). *Asterisks* indicate significant differences (*P < 0.05, **P < 0.01).

## Results

### Cloning and sequence analysis of six heat-responsive *MYB* genes in wheat

**Fig. 1:**
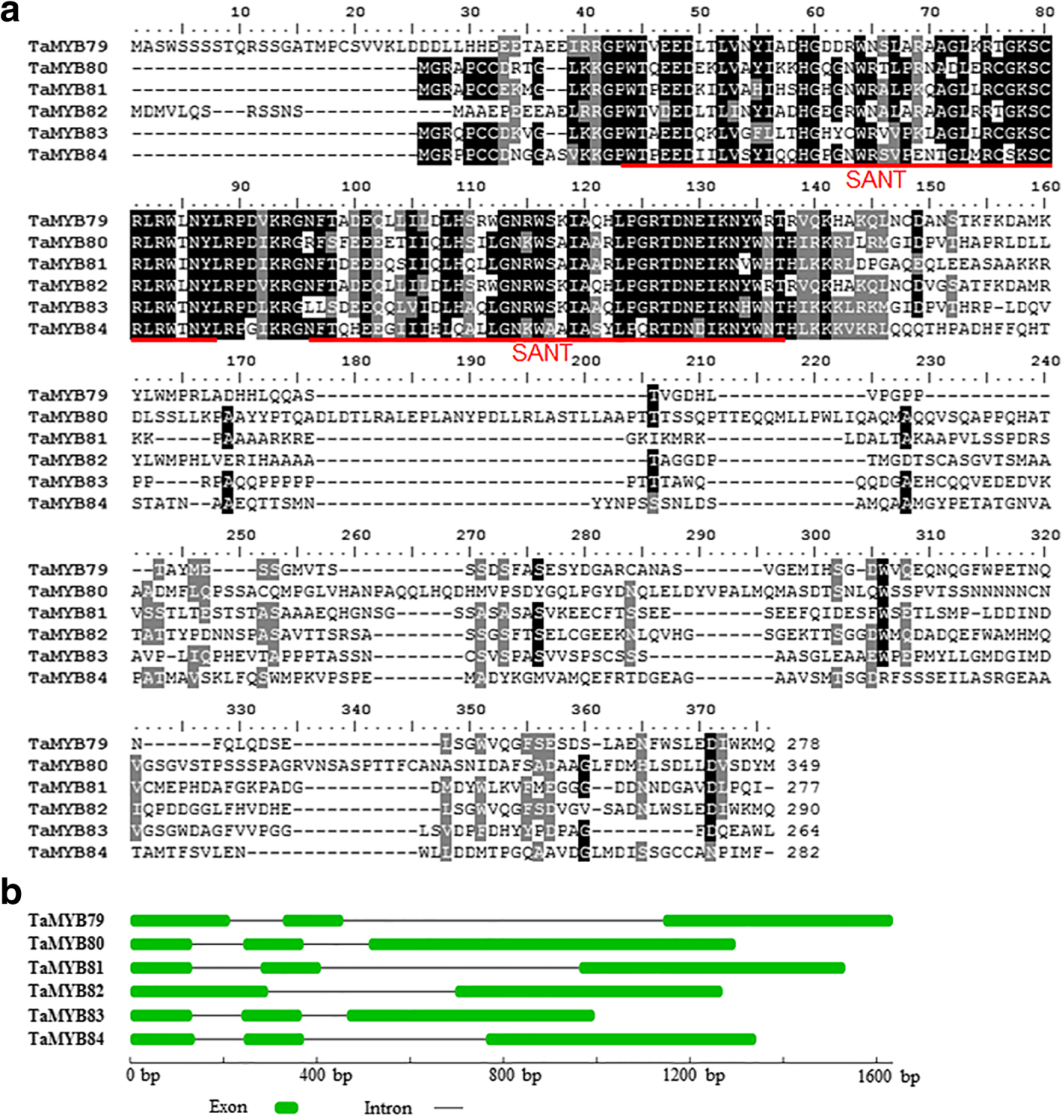
The protein sequence alignment and exon-intron structure of six wheat MYB genes responsive to heat. **a** The protein sequence alignment of wheat MYB proteins. Each of the six TaMYB proteins contained two SANT domains (*underlined in red*). **b** The gene structure of heat-responsive *TaMYBs*. Green boxes and black lines represent exons and introns, respectively. The scale bars on the line below indicate gene sizes

We recently performed high-throughput transcriptome sequencing of wheat (TAM107) seedlings grown under normal conditions and subjected to heat stress for 6 h [10]. By scanning the transcriptome data, we identified multiple MYB TF genes up-regulated (fold change >2) by heat stress. Among these genes, we selected six novel heat-responsive MYB genes and isolated their genomic and coding sequences by PCR from wheat cultivar TAM107. According to Bi et al. [37], wheat MYB genes are named after *TaMYB78*; therefore, these six wheat genes were named *TaMYB79–84*. Details of the six cloned wheat MYB genes, including name, accession number, chromosomal location, molecular weight, and isoelectric point (*p*I), are summarized in Table 1. The domain structure analyses revealed that all six MYB sequences were in the R2R3-MYB subfamily, and each contained two adjacent, highly conserved SANT DNA-binding domains, a characteristic domain of MYB TFs and localized in the N-terminal portion of the proteins (Fig. 1a). The gene structure of the six wheat MYB genes was analyzed by pairwise comparison of their full-length cDNA and genomic DNA sequences. *TaMYB79*, *TaMYB80*, *TaMYB81*, *TaMYB83*, and *TaMYB84* genes contained two introns and three exons, and *TaMYB82* contained only one intron and two exons (Fig. 1b).

**Table 1:** Cloned six wheat *MYB* genes

Cloned genes	Accession number	Coding sequence	Chromosomal	Molecular	Isoelectric
		length (bp)	location	weight	point (*p*I)
*TaMYB79*	KY475607	837	5AS	31.7	5.47
*TaMYB80*	KY475608	1050	2AS	38.6	5.98
*TaMYB81*	KY475609	834	2AL	30.6	6.16
*TaMYB82*	KY475610	873	4BS	32.3	5.46
*TaMYB83*	KY475611	795	7BS	29.1	5.98
*TaMYB84*	KY475612	849	4AL	31	7.04

The chromosomal location of cloned genes was obtained by blasting against the International Wheat Genome Sequencing Consortium (IWGSC) database. The molecular weight and isoelectronic point (*p*I) were calculated using the ProtParam tool (http://web.expasy.org/protparam/)

### Nuclear localization of the six TaMYB proteins

**Fig. 2:**
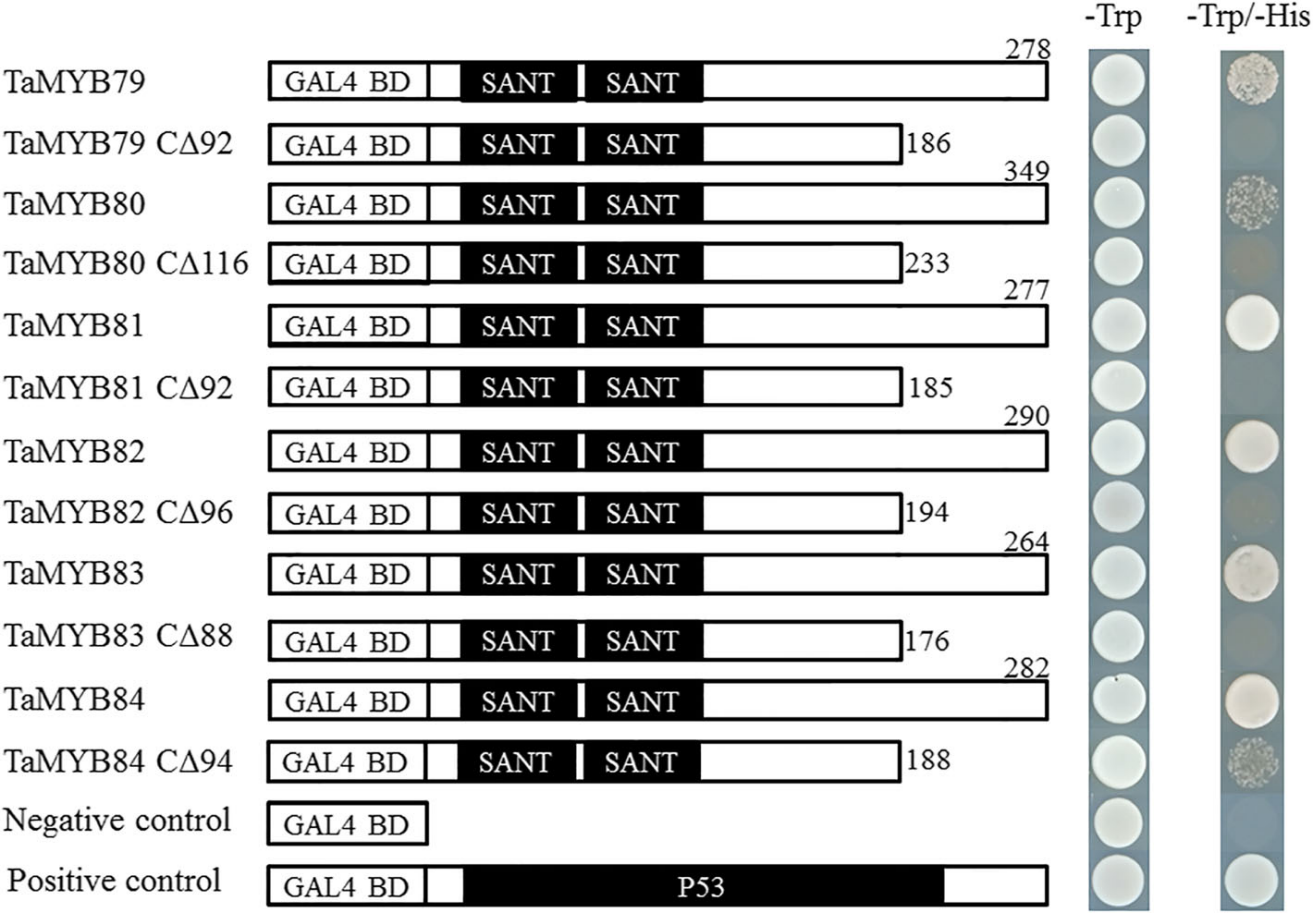
Subcellular localization of TaMYB proteins. The 35S::TaMYB79-GFP, 35S::TaMYB80-GFP, 35S::TaMYB81-GFP, 35S::TaMYB82-GFP, 35S::TaMYB83-GFP and 35S::TaMYB84-GFP constructs and the 35S::GFP control vector were transiently expressed in wheat protoplasts. Results were visualized under confocal microscopy 16 h after transformation. Bars = 20 μm

To provide evidence of the role of all six selected TaMYB proteins in transcriptional regulation, we determined their subcellular localizations. The full-length sequence of each TaMYB was fused to the *green fluorescent protein* (*GFP*) gene sequence in the pJIT163-GFP vector. Each of the resulting constructs, designated TaMYB79-GFP, TaMYB80-GFP, TaMYB81-GFP, TaMYB82-GFP, TaMYB83-GFP, and TaMYB84-GFP, was transiently expressed in wheat protoplasts. As shown in Fig. 2, the control GFP, product of the construct 35S::GFP, was uniformly distributed throughout the mesophyll cell protoplast, whereas TaMYB fusion proteins were primarily localized in the nucleus.

### The C-terminal region of each TaMYB has transcriptional activation activity

**Fig. 3:**
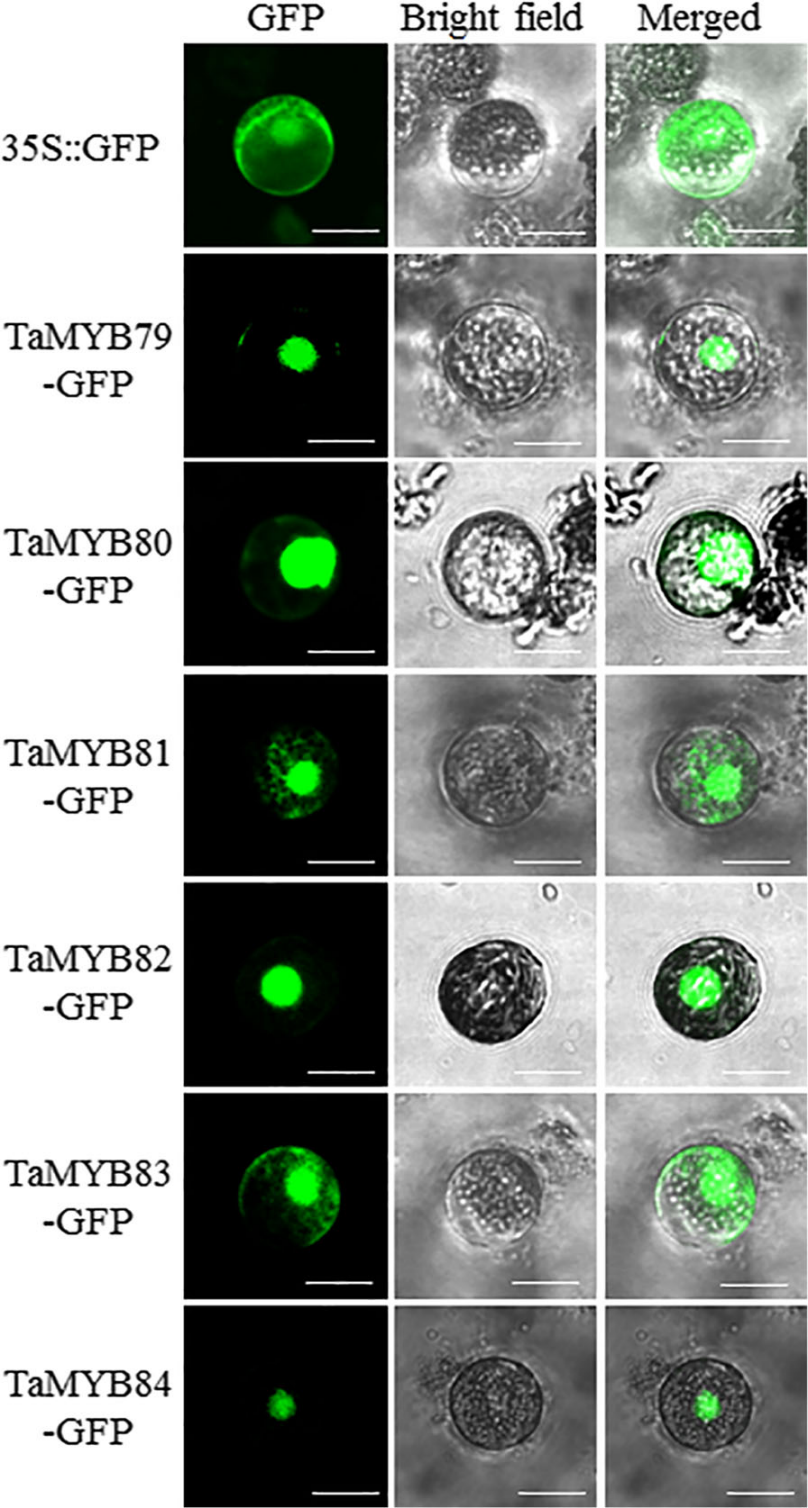
Transcriptional activity assay of the full-length and truncated heat-responsive TaMYB proteins in yeast. The length of each TaMYB protein and respective protein truncation are indicated. Fusion proteins of the GAL4 DNA-binding domain and full-length TaMYBs or truncated TaMYBs with deletion of X C-terminal amino acid residues (CΔX) were expressed in yeast strain AH109. The empty pGBKT7 vector and the pGBKT7-P53 construct were used as negative and positive controls, respectively. Equal amounts of liquid cultures of the transformed yeast cells were dropped onto SD plates containing no tryptophan (Trp) and onto SD plates without Trp and histidine (His)

To test whether TaMYBs had transcriptional activation activity, full-length coding sequences of TaMYBs were fused in-frame to the GAL4 DNA-binding domain of the pGBKT7 vector, and the fusion constructs were transformed into the yeast strain AH109. As shown in Fig. 3, the yeast transformants containing each of the pGBKT7-TaMYB plasmids or the positive control plasmid grew on an SD/−Trp-His plate, whereas yeasts harboring the pGBKT7-unloaded plasmid (negative control) did not grow.

According to a previous report, the C-terminal region of an MYB TF is required for transcriptional activation activity [38]. To identify the C-terminal region of the investigated TaMYBs responsible for transcriptional activation, CΔX (without the C-terminal X amino acids) regions of TaMYBs were fused to the GAL4 BD (Fig. 3). Yeasts containing CΔX regions of TaMYBs did not grow on an SD/−Trp-His plate, with the exception of TaMYB84, which led to weak growth of yeast.

### Phylogenetic analysis of the six wheat MYBs

**Fig. 4:**
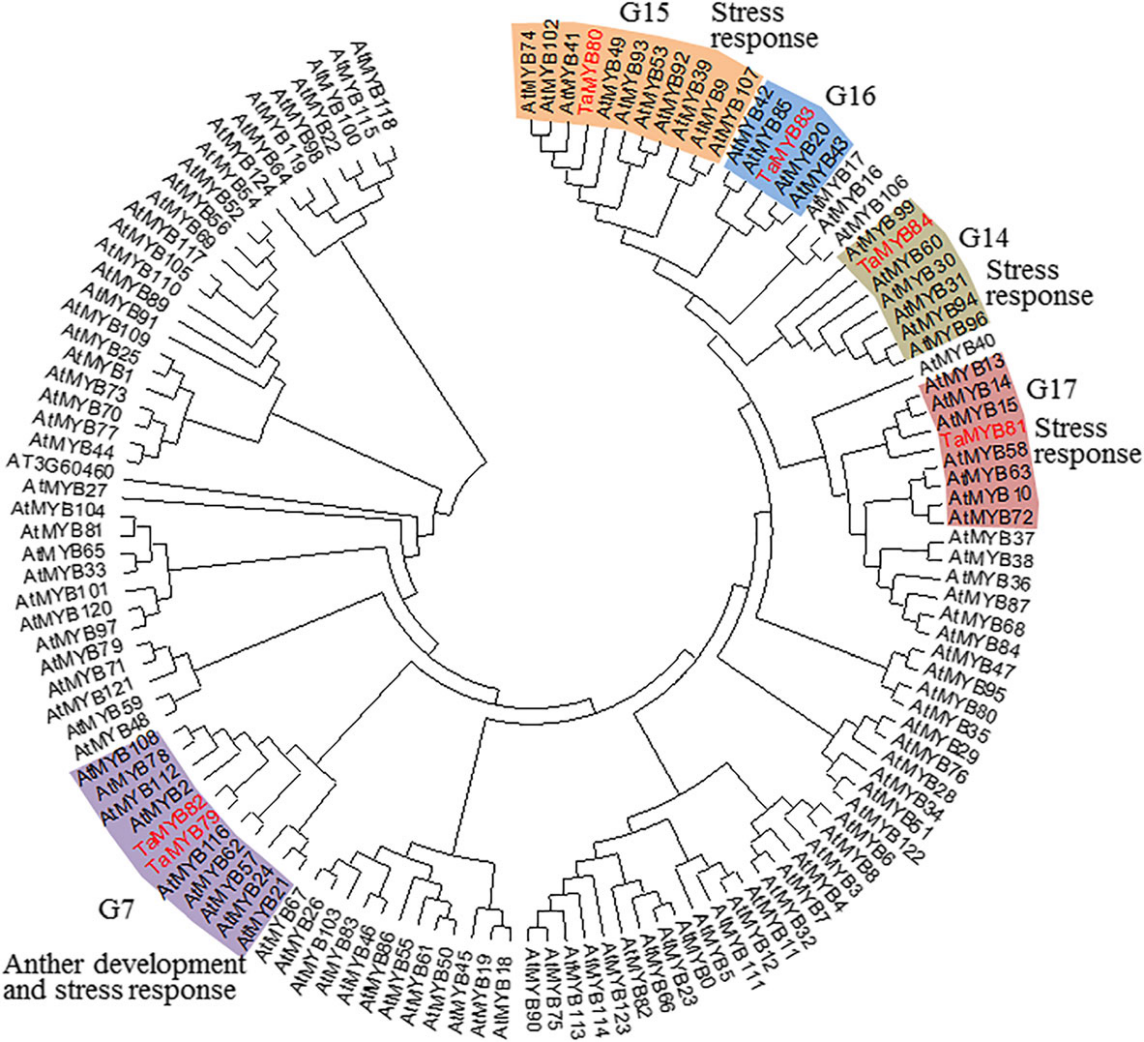
Phylogenetic relationships of the six wheat MYBs and *Arabidopsis* R2R3-MYB proteins. The complete amino acid sequences of TaMYB79–84 and 126 *Arabidopsis* R2R3-MYB proteins were aligned by ClustalW. The phylogenetic tree was constructed using MEGA6.0 with the neighbor-joining method. The bootstrap value was 1000 replicates. Each group containing a wheat MYB or wheat MYBs is indicated by a specific color. Wheat MYBs are highlighted in red

To further predict the functions of the six R2R3-MYB TFs, the evolutionary relationships of 132 members, including 126 *Arabidopsis* R2R3-MYB protein sequences and the 6 products of *TaMYB* genes cloned in this study, were inferred using the neighbor-joining method in MEGA6 (Fig. 4). The TaMYB TFs were clustered with different clades (indicated by red in Fig. 4), representing mostly *Arabidopsis* proteins with previously determined roles in responses to abiotic stress [39]. The G7 subgroup that contained TaMYB79, TaMYB82 and *Arabidopsis* proteins AtMYB2, AtMYB24, AtMYB78, AtMYB108, and AtMYB112 is involved in the regulation of anther development and responses to environmental signals [40, 41]. TaMYB80 was grouped into the G15 subgroup with AtMYB41, AtMYB74, and AtMYB102, which is involved in ABA-mediated responses to abiotic stress [14, 16]. TaMYB81 and TaMYB84 were in the G17 and G14 subgroups, respectively, with members that participate in abiotic stress responses in *Arabidopsis* [17, 42].

### Expression of the six *TaMYBs* in different wheat tissues

**Fig. 5:**
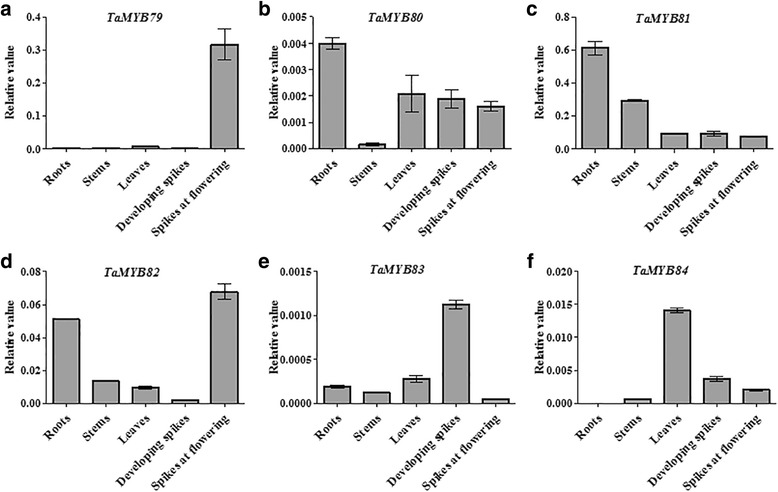
Tissue-specific expression patterns of the TaMYB79 (**a**), TaMYB80 (**b**), TaMYB81 (**c**), TaMYB82 (**d**), TaMYB83 (**e**), and TaMYB84 (**f**). Total RNA was isolated from roots, stems, leaves, developing spikes, and spikes at flowering. The wheat *β-actin* gene was used as the internal reference. Bars show standard deviations of three replicates

Tissue-specific gene expression is generally associated with specific biological functions. Therefore, we characterized the expression patterns of the six *TaMYBs* in different tissues, including roots, stems, leaves, developing spikes, and spikes at flowering, using RT-qPCR. The six *TaMYBs* differed greatly in their expression profiles (Fig. 5). *TaMYB79* showed a high level of expression in spikes at flowering, but very low levels of expression in other tissues (Fig. 5a). The highest levels of *TaMYB80* and *TaMYB81* expression were in the roots (Fig. 5b, c). *TaMYB82* transcripts were abundant in roots and spikes at flowering, but expression levels of this gene in stems, leaves, and developing spikes were much lower (Fig. 5d). For *TaMYB83*, the highest level of transcripts was in developing spikes, with a moderate level in leaves (Fig. 5e), whereas *TaMYB84* had the highest expression level in leaves, with low transcript levels in other tissues (Fig. 5f).

### Expression of the six *TaMYBs* under different abiotic stresses and exogenous ABA treatment

**Fig. 6:**
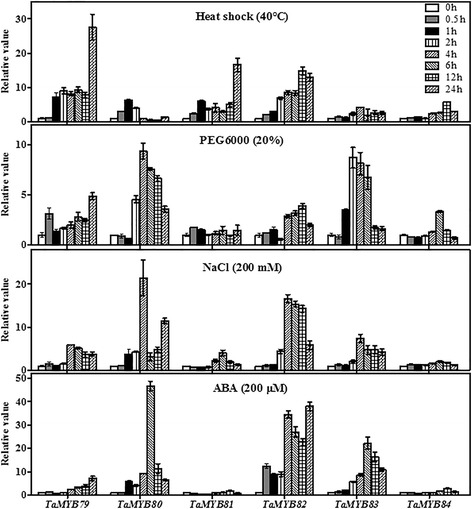
RT-qPCR assessment of the expression of six wheat *MYBs* under different stress conditions. Total RNAs were extracted from 7-day-old wheat seedlings subjected to either heat shock at 40 °C, 20% PEG 6000, 200 mM NaCl or 200 μM ABA treatment for the indicated time periods. The expression levels of genes at 0 h (not treated) were set to “1”. The wheat *β-actin* gene was used as the internal reference. Bars represent standard deviations of three biological replicates. X-axes show the time courses of abiotic stress treatments for each gene. Y-axes indicate the scales of the relative expression levels

The responses of *TaMYBs* to heat, drought, high salinity and exogenous ABA treatments were analyzed by RT-qPCR. As shown in Fig. 6, the expression of the six *TaMYBs* was dramatically up-regulated by heat stress (40 °C). The mRNA of four genes, *TaMYB79*, *TaMYB80*, *TaMYB81*, and *TaMYB82*, rapidly accumulated after 1 h of heat treatment. The expression of all *TaMYBs*, except for *TaMYB81*, was obviously up-regulated in wheat seedlings during the PEG treatment mimicking drought. Under the high salt treatment (200 mM), the transcripts of the *TaMYBs* were clearly enhanced after 4 h of treatment, with the exception of *TaMYB84*. In response to exogenous ABA, the expression of *TaMYB79*, *TaMYB80*, *TaMYB82*, and *TaMYB83* was significantly up-regulated, whereas *TaMYB84* showed weak up-regulation and the number of *TaMYB81* transcripts initially decreased and then slightly increased. Overall, each *TaMYB* responded differently to the various treatments.

### *TaMYB80* confers heat and drought tolerance in transgenic *Arabidopsis*

**Fig. 7:**
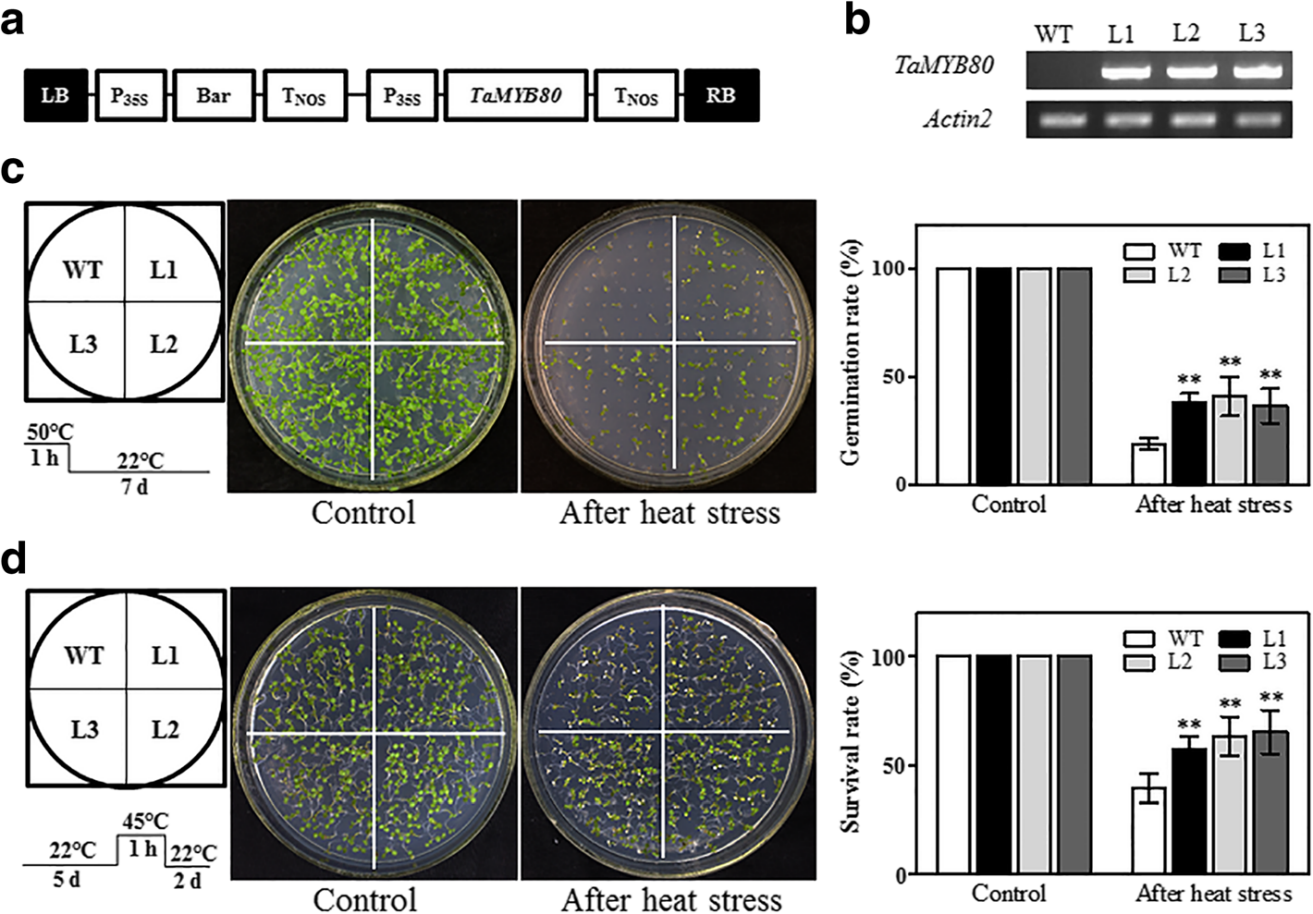
Overexpression of *TaMYB80* confers heat tolerance to transgenic *Arabidopsis* plants. **a** Schematic diagram of the *TaMYB80* overexpression construct driven by the 35S promoter. **b** Analysis of *TaMYB80* transcript levels in WT *Arabidopsis* and transgenic lines (L1*,* L2, and L3). Total RNA was isolated from 1-week-old plants. *Actin2* was used as the control gene. **c** Germination rates (%) of *TaMYB80*-overexpression plants after treatment with heat stress. Seeds of WT and transgenic lines were sterilized and subjected to 50 °C treatment for 1 h and then germinated on 1% agar plates. The plates were placed at ambient conditions (22 °C) for 7 d before photographs were taken. At least 50 seeds were assessed per replicate for five independent replicates. **d** Survival rates (%) of WT and transgenic seedlings after treatment with heat stress. Five-day-old seedlings were heat-treated at 45 °C for 1 h before returning to 22 °C to grow for two days, and then photographs were taken and the survival rates were calculated. More than 40 plants of each line were used. Bars represent standard deviations

As described above, the *TaMYB80* gene was strongly induced by multiple abiotic stresses. To confirm the functions of *TaMYB80* in the abiotic stress response, we generated transgenic *Arabidopsis* plants overexpressing *TaMYB80* driven by the *CaMV 35S* promoter (Fig. 7a). Three T_3_ homozygous transgenic lines (L1, L2, and L3) containing a single insertion of the *TaMYB80* were selected for phenotypic analyses. The transcript levels of *TaMYB80* expression in these lines were determined by RT-PCR (Fig. 7b).

The germination rate was not significantly different between the WT and the transgenic lines under normal temperature (22 °C) conditions. However, all three transgenic lines exhibited significantly higher germination percentages than the WT plants when seeds were germinated after 1 h of exposure at 50 °C on 1% agar plates (Fig. 7c). For the survival assay under heat stress, 5-day-old seedlings of WT and transgenic lines were heat-treated at 45 °C for 1 h followed by 2 days of recovery under normal conditions (22 °C). The survival rates of all transgenic lines (57%, 63% and 65%) were significantly higher than that of the WT plants (40%; Fig. 7d).

**Fig. 8:**
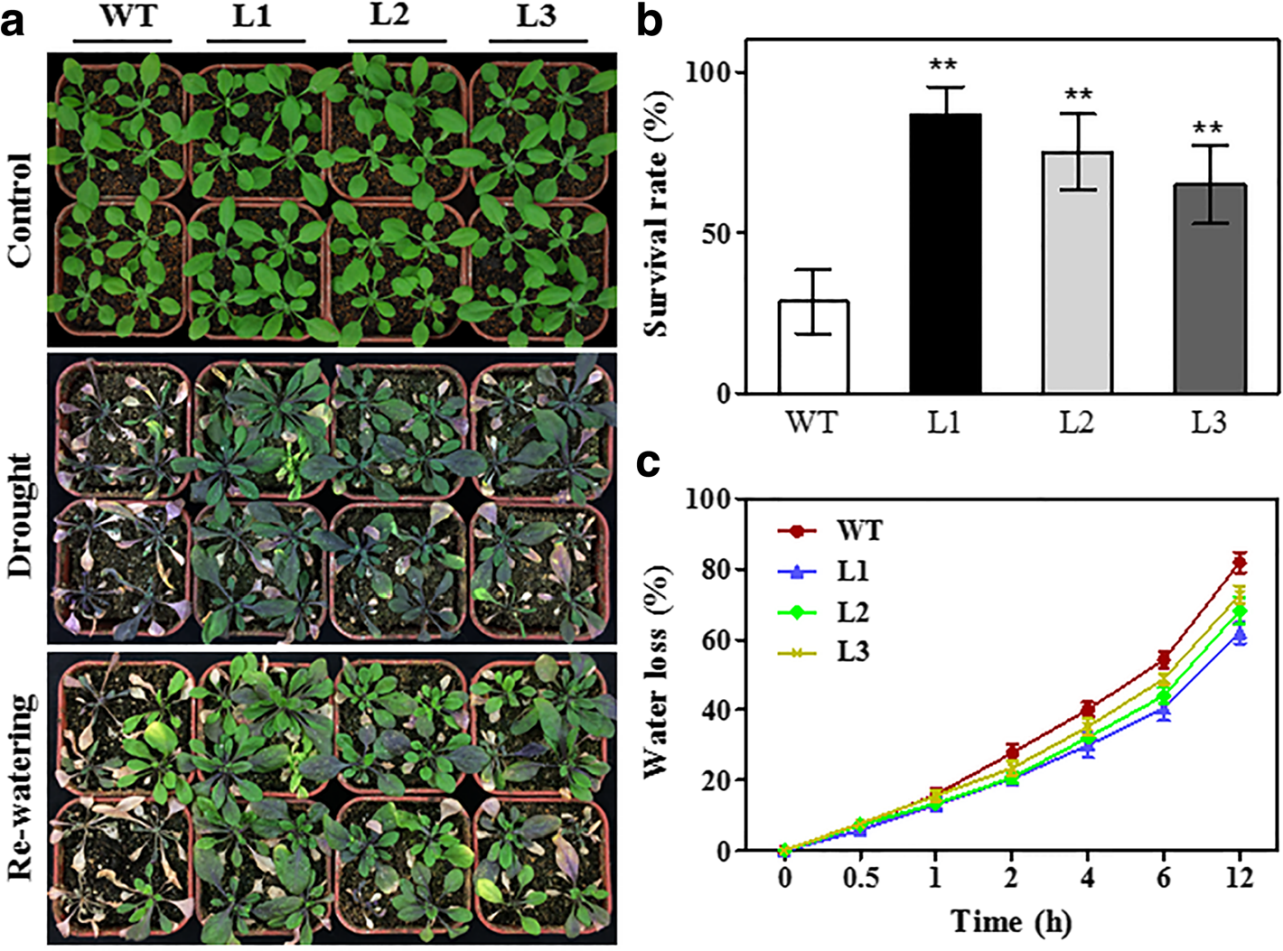
Phenotypic differences between transgenic lines overexpressing *TaMYB80* and WT plants under drought treatment. **a** Photographs showing plants before and after dehydration stress. Four-week-old seedlings of WT and three transgenic lines were subjected to drought stress treatment by withholding irrigation for 14 days, followed by resumed watering and plant growth for another two days. Three independent experiments were conducted. **b** Survival rates of WT and three transgenic lines after drought treatment. Bars represent standard deviations (*n* = 60) of three biological replicates. **c** Water loss from detached leaves of 4-week-old plants measured at room temperature. Error bars represent the standard deviations of three replicates with 5 seedlings per replicate

To validate that the *TaMYB80* gene was also involved in drought stress tolerance, 14-day-old WT and transgenic plants were grown in soil and subjected to a water-withholding treatment for 14 days, followed by re-watering for 3 days. Under normal growth conditions (22 °C), the transgenic lines showed no obvious difference in performance compared with the WT plants (Fig. 8a). However, after withholding water for 14 days, the WT leaves were severely dehydrated, whereas the transgenic plants showed much less wilting (Fig. 8a). After the 3-day recovery, only 30% of the WT plants survived, whereas more than 70% of the transgenic plants survived in lines L1, L2, and L3 (Fig. 8b). Additionally, under drought stress, the transgenic plants showed lower rates of water loss than the WT plants (Fig. 8c).

### Overexpression of *TaMYB80* increases cellular ABA content and expression of ABA-dependent stress-related genes

**Fig. 9:**
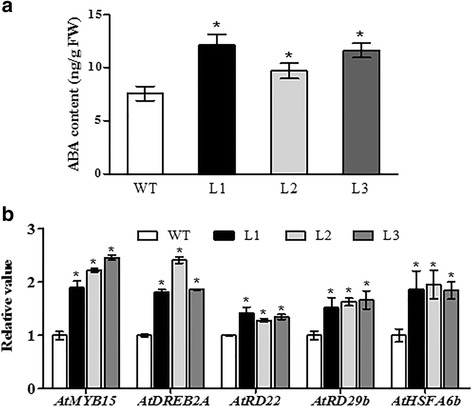
Endogenous ABA content and expression of stress-related genes of WT and transgenic plants under normal conditions. **a** ABA levels were determined in 21-day-old seedlings. FW, Fresh weight. **b** Expression of ABA-dependent stress-related genes in WT and *TaMYB80* transgenic lines. The total RNA was extracted from 21-day-old seedlings. The *Arabidopsis Actin2* gene was used as the internal control. The gene expression in WT was regarded as standard, and transgenic lines (L1, L2, and, L3) values were compared with it. Error bars represent the standard deviations of three biological replicates

As shown above, overexpression of the *TaMYB80* gene in *Arabidopsis* increased drought tolerance and reduced water loss. ABA plays a role in decreasing water loss via the induction of stomatal closure and some other mechanisms [43, 44]. Therefore, we hypothesized that the transgenic plants might have elevated amounts of endogenous ABA in the absence of stress. To test this hypothesis, we measured the content of endogenous ABA using a UPLC–MS/MS system. The results of three independent experiments showed that levels of ABA in transgenic plants (L1, L2, L3) were increased compared with that of WT plants (Fig. 9a). These data clearly demonstrate that the overexpression of the *TaMYB80* gene increases cellular ABA levels.

To investigate the molecular basis of the stress tolerance provided by the *TaMYB80* gene for transgenic *Arabidopsis*, we analyzed the expression of several stress-related genes in three independent transgenic lines (L1, L2, and L3) and WT plants via RT-qPCR. We found that the expression levels of several genes positively regulated by ABA, including *AtMYB15*, *AtHSFA6b*, *AtDREB2A*, *AtRD22*, and *AtRD29b*, were upregulated in the transgenic lines (Fig. 9b).

## Discussion

High temperature negatively affects plant growth and crop productivity. Cloning and functional characterization of heat responsive genes in crops is the first step in providing valuable information for crop thermotolerance breeding. MYB transcription factors constitute one of the largest transcription factor families, and some members of the family play crucial roles in the regulation of stress responses, particularly in response to high temperatures [26, 28]. However, data on heat-responsive MYB genes in wheat are limited [10]. In this study, six putative heat-induced MYB genes were cloned from a heat and drought tolerant wheat cultivar TAM107. The putative functions of the six *TaMYBs* were investigated by phylogenetic analysis and expression profile analyses in different wheat tissues and under multiple stress treatments. We also showed that constitutive overexpression of *TaMYB80* in transgenic *Arabidopsis* improved plant tolerance to high temperatures and drought.

Transactivation assays in yeast revealed that the tested TaMYBs all had transcriptional activation activity. In particular, TaMYB81, TaMYB82, TaMYB83, and TaMYB84 showed strong transcriptional activation, whereas weak transcriptional activation was observed for TaMYB79 and TaMYB80 (Fig. 3). Additionally, yeasts containing CΔX regions of individual TaMYBs (except TaMYB84) could not grow on an SD/−Trp-His plate, suggesting that the excised C-terminal region of each tested TaMYB (except TaMYB84) was essential for the transcriptional activation of a gene. In the case of truncated TaMYB84, clear but weak growth was observed (Fig. 3), and so a significant part of the AD was in the C-terminal region.

As previously reported, 126 *Arabidopsis* R2R3-MYB TFs are divided into 17 groups based on complete amino acid sequence, with the group often comprising TFs with similar functions [39]. Five wheat MYB proteins characterized in this study were clustered into four *Arabidopsis* groups that contained products of stress-responsive genes. The remaining TaMYB83 was clustered in group G16, which is not a stress response group but shares a close evolutionary relationship with AtMYB20, which is involved in stress response (Fig. 4). Previous studies have indicated that the overexpression of *AtMYB20* in transgenic *Arabidopsis* plants increased salt tolerance [45], implying that TaMYB83 may also play a role in stress response of wheat. Overall, the results suggested that the six TaMYB proteins are involved in wheat responses to abiotic stresses.

Gene expression patterns are indications of gene functions [46]. In this study, tissue- and developmental stage-specific expression of *TaMYB* genes suggested diverse functions of their products. Preferred or specific expression of *TaMYB79* in spikes at flowering, *TaMYB81* in roots, *TaMYB83* in developing spikes, and *TaMYB84* in leaves indicated that these *MYBs* might be involved in the development of different organs and tissues. Likewise, expression analysis of the six *TaMYBs* in leaves of seedlings under different stresses showed that each of the tested *TaMYBs* differentially responded to 2–4 abiotic stresses and treatments, indicating the involvement of these MYB TFs in the physiological processes associated with multiple stress responses in wheat. According to previous studies, many MYB TFs respond to abiotic stresses via the ABA-mediated signaling pathway. For example, *AtMYB2* regulates the ABA-dependent expression of salt- and dehydration-responsive genes, and its interaction with a calmodulin increases salt tolerance [47]. Another MYB TF, *AtMYB96*, mediates ABA signals via *RD22* in plant resistance responses to water deficit by reducing stomatal openings [48]. By contrast, *AtMYB20*, a negative regulator of ABA signaling, increased plant salt tolerance by down-regulating the expression of *PP2C* genes [45]. Gene expression analysis showed that *TaMYB79*, *TaMYB80*, *TaMYB82*, and *TaMYB83* were strongly induced under ABA treatment (Fig. 4), suggesting that each of these genes may be part of an ABA-dependent signaling transduction pathway in response to abiotic stress. Exogenous ABA levels did not affect the expression patterns of the other two heat-inducible *TaMYB* genes (*TaMYB81* and *TaMYB84*).

Ectopic expression of wheat genes in the model plant *Arabidopsis* is a fast and effective approach to investigate gene functions [49]. In the present study, transgenic *Arabidopsis* plants overexpressing *TaMYB80* were generated; these plants showed increased tolerance to heat and drought stresses and had higher ABA content (Figs. 7, 8 and 9a). As shown in Fig. 8, plant growth and survival increased significantly for the transgenic plants during drought treatment. All WT plants showed severe wilting, and some even died, whereas the rosette leaves of some of the transgenic plants remained green. Abogadallah et al. found that the overexpression of *HARDY* improved the growth of *Trifolium alexandrinum* under drought stress, which may have resulted from higher rates of photosynthesis [50]. Thus, the rates of photosynthesis in *TaMYB80* transgenic plants could have been higher than those in WT plants, resulting in the increased level of drought tolerance. Further studies are required to test this hypothesis. ABA has an important role in the response of plants to water deficit by regulating stomatal closure and the expression of stress-related genes [51]. For example, in transgenic plants overexpressing *AtLOS5*, *AtHSPR*, or *OsASR5* genes, stomatal apertures decrease via increased ABA content in response to drought stress [44, 52, 53]. Previously, ABA-dependent stomatal closure was thought to be disadvantageous for plant acclimation to heat stress because closure could prevent leaf cooling via transpiration [54]; however, increases in endogenous ABA level and ABA-dependent stomatal closure have been reported to participate in protection from heat damage [54–57]. For example, a recent study demonstrated that the RING finger ubiquitin E3 ligase OsHTAS positively regulated ABA biosynthesis and induced stomatal closure during heat stress, consequently increasing the heat tolerance of rice [57]. Additionally, temporal-spatial interaction between ABA and reactive oxygen species signals plays a key role in the regulation of systemic acquired acclimation of plants to heat stress [58]. Thus, we hypothesized that the increased tolerance of *TaMYB80* overexpression plants to heat and drought stresses was most likely due to the increased level of endogenous ABA.

Further study revealed that increases in endogenous ABA levels modified the expression of several ABA-dependent stress-related genes, including *AtMYB15*, *AtHSFA6b*, *AtDREB2A*, *AtRD22*, and *AtRD29b* (Fig. 9b). *AtMYB15* increases the expression of genes involved in ABA biosynthesis and when overexpressed in transgenic plants, improves salt and drought tolerance [17]. *HSFA6b* plays a primary role in an ABA-mediated signaling network related to heat stress responses [59], and *DREB2A* is a crucial regulatory element involved in drought stress response [60]. *RD22* and *RD29b* encode low molecular hydrophilic proteins and have a cellular protection function [17]. In this study, the expression levels of these genes were significantly higher in the transgenic plants than in the WT plants. This result suggested that the overexpression of *TaMYB80* enhanced the expression levels of ABA-dependent stress-related genes, thereby conferring heat and drought tolerance in *Arabidopsis*. Future studies on the possible roles of *TaMYB80* in ABA biosynthesis and/or ABA signaling pathways would be of interest.

## Conclusions

In this study, we identified and cloned six heat-induced MYB genes. All the genes were localized in the nucleus and were demonstrated to function as transcriptional activators. The ADs of all six MYB TFs were localized at the C-terminus of the molecules. Phylogenetic analysis assigned all six proteins to clades containing stress-related MYB TFs. Different expression profiles of *MYB* genes were revealed in various wheat tissues and under multiple stress treatments. We demonstrated that the overexpression of *TaMYB80* in transgenic *Arabidopsis* increased plant tolerance to high temperatures and drought. Our study provides useful information for breeders and genetic engineers working to improve the heat and drought tolerance of wheat.

## Additional file

Additional file 1: Table S1. Primer sequences used in this study (DOCX 24 kb)

## Abbreviations

ABA: Abscisic acid
AD: Activation domain
BD: Binding domain
CaMV: Cauliflower mosaic virus
DREB: Dehydration-responsive element-binding protein
GFP: Green fluorescent protein
HSF: Heat shock factor
PEG: Polyethylene-glycol
PP2C: Protein phosphatase 2C
RD: Responsive to dehydration
RT-qPCR: Reverse transcription quantitative real-time polymerase chain reaction
SD: Synthetic defined
TFs: Transcription factors
UPLC–MS/MS: Ultra-performance liquid chromatography tandem mass spectrometry
WT: Wild-type
