# Supplementary material for: Characterization of wheat MYB genes responsive to high temperatures
Source: BMC Plant Biol. 2017 Nov 21;17:208. doi: 10.1186/s12870-017-1158-4 (PMC5696766; doi:10.1186/s12870-017-1158-4)
Supplement: Additional file 1: Table S1. — Primer sequences used in this study (DOCX 24 kb) [file 12870_2017_1158_MOESM1_ESM.docx]

**Table S1.** Primer sequences used in this study

| **Reactions** | **Primer name** | | **Sequence information** | | **Enzyme**  **site** | |
| --- | --- | --- | --- | --- | --- | --- |
| Gene cloning | | TaMYB79-F | | ATGGCGTCATGGTCATCGTC | |  |
|  | | TaMYB79-R | | CACCATGCCGACCATAAATCG | |  |
|  | | TaMYB80-F  TaMYB80-R  TaMYB81-F  TaMYB81-R | | GCCCAATCCGTCTCTACTCTA  AGGTGGAACCCGTACTTGTAT  GACTCCTCCGCATCTGCATC  CGATCAATCAGGAGCCAAGTC | |  |
|  | | TaMYB82-F | | TCCTCCACTAGCTACGTCGTC | |  |
|  | | TaMYB82-R | | CCATCCTCACTCCGATGTATATG | |  |
|  | | TaMYB83-F  TaMYB83-R  TaMYB84-F  TaMYB84-R | | GATCGAGCCATCACTCAGTC  GTCTATGTGCATTGCTGCTG  CTGGCGGCACCAACGACAT  GATTGGCCACCAAGAGTGGTAAG | |  |
| Transcriptional | | TaMYB79-TF | | TGGCCATGGAGGCCGAATTCATGGCGTCATGGTCA | | *Eco R*I |
| activation | | TaMYB79-TR | | CGACGGATCCCCGGGAATTCTCATTGCATCTTCCA | | *Eco R*I |
|  | | TaMYB79-CΔR | | CGACGGATCCCCGGGAATTCTCAACCAACTGTAGA | | *Eco R*I |
|  | | TaMYB80-TF | | TGGCCATGGAGGCCGAATTCATGGGGCGCGCGCCGT | | *Eco R*I |
|  | | TaMYB80-TR | | CGACGGATCCCCGGGAATTCTTACATGTAGTCGCTCAC | | *Eco R*I |
|  | | TaMYB80-CΔR | | CGACGGATCCCCGGGAATTCCGAGCTGGGCTGCAAG | | *Eco R*I |
|  | | TaMYB81-TF | | TGGCCATGGAGGCCGAATTCATGGGGAGGGCTCCG | | *Eco R*I |
|  | | TaMYB81-TR | | CGACGGATCCCCGGGAATTCCTAAATCTGCGGTAA | | *Eco R*I |
|  | | TaMYB81-CΔR | | CGACGGATCCCCGGGAATTCTCACGTGGACTCGGT | | *Eco R*I |
|  | | TaMYB82-TF | | TGGCCATGGAGGCCGAATTCATGGATGTGGTGCTG | | *Eco R*I |
|  | | TaMYB82-TR | | CGACGGATCCCCGGGAATTCTCATTGCATCTTCCA | | *Eco R*I |
|  | | TaMYB82-CΔR | | CGACGGATCCCCGGGAATTCTCAGTTGTTCTCGGG | | *Eco R*I |
|  | | TaMYB83-TF | | TGGCCATGGAGGCCGAATTCATGGGGAGGCAGCCG | | *Eco R*I |
|  | | TaMYB83-TR | | CGACGGATCCCCGGGAATTCTTACAGCCAGGCTTC | | *Eco R*I |
|  | | TaMYB83-CΔR | | CGACGGATCCCCGGGAATTCTCACGGCTGGATCAG | | *Eco R*I |
|  | | TaMYB84-TF | | TGGCCATGGAGGCCGAATTCATGGGGAGGCCGCCA | | *Eco R*I |
|  | | TaMYB84-TR | | CGACGGATCCCCGGGAATTCTCAAAACATGATGGG | | *Eco R*I |
|  | | TaMYB84-CΔR | | CGACGGATCCCCGGGAATTCTCACTCCGGCGATGG | | *Eco R*I |
| RT-qPCR | | TaMYB79-qF | | TGATGGTGCACGCTGTG | |  |
|  | | TaMYB79-qR | | CCATAAATCGTATATGCACGTGA | |  |
|  | | TaMYB80-qF | | CAGATGCTCCTCCCTTGG | |  |
|  | | TaMYB80-qR | | GTGATCCTGGTGTAGTTGC | |  |
|  | | TaMYB81-qF | | AGGAGTTCCAGATCGACGAG | |  |
|  | | TaMYB81-qR | | TTGAGCCAGTAGTCCATGTC | |  |
|  | | TaMYB82-qF | | TTCGTTCACATCGGAGCTCT | |  |
|  | | TaMYB82-qR | | GCATCTTCCATATGTCCTCG | |  |
|  | | TaMYB83-qF | | TCAAGAACCACTGGAACACC | |  |
|  | | TaMYB83-qR | | TCCTCATCCTCCACTTGCT | |  |
|  | | TaMYB84-qF | | GTGGCCATGCAGGAGTTC | |  |
|  | | TaMYB84-qR | | CATGTCGTCGAGCAACCAG | |  |
|  | | β-actin-F | | GACCGTATGAGCAAGGAGAT | |  |
|  | | β-actin-R | | CAATCGCTGGACCTGACTC | |  |
|  | | AtMYB15-qF | | ATGGGAAGAGCTCCATGCTGTG | |  |
|  | | AtMYB15-qR | | CTAGAGCCCGGCTAAGAGATC | |  |
|  | | AtDREB2A-qF | | GACCTAAATGGCGACGATGT | |  |
|  | | AtDREB2A-qR | | TCGAGCTGAAACGGAGGTAT | |  |
|  | | AtHSFA6b-qF | | GTGATGAAAGTGGTTATGGGAATG | |  |
|  | | AtHSFA6b-qR | | TCCGACATCTCGAATTCAGACAT | |  |
|  | | AtRD29b-qF | | CCGACAAGAGGTGATGTGAAAGTAG | |  |
|  | | AtRD29b-qR | | GTGTAACCTAGCTTTGAGGCAACG | |  |
|  | | AtRD22-qF | | ACTTGGTAAATATCACGTCAGGGCT | |  |
|  | | AtRD22-qR | | CTGAGGTGTTCTTGTGGCATACC | |  |
| Subcellular | | TaMYB79-SF | | AGCCCAAGCTTATGGCGTCATGGTCA | | *Hind* III |
| localization | | TaMYB79-SR | | GCTCTAGATTGCATCTTCCAAATG | | *Xba* I |
|  | | TaMYB80-SF | | AGCCCAAGCTTATGGGGCGCGCGCCGT | | *Hind* III |
|  | | TaMYB80-SR | | GCTCTAGACATGTAGTCGCTCACATC | | *Xba* I |
|  | | TaMYB81-SF | | AGCCCAAGCTTATGGGGAGGGCTCC | | *Hind* III |
|  | | TaMYB81-SR | | GCTCTAGAAATCTGCGGTAAGTC | | *Xba* I |
|  | | TaMYB82-SF | | AGCCCAAGCTTATGGATATGGTGCTGCA | | *Hind* III |
|  | | TaMYB82-SR | | GCTCTAGATTGCATCTTCCATATGTC | | *Xba* I |
|  | | TaMYB83-SF | | AGCCCAAGCTTATGGGGAGGCAGCC | | *Hind* III |
|  | | TaMYB83-SR | | GCTCTAGACAGCCAGGCTTCTTG | | *Xba* I |
|  | | TaMYB84-SF | | AGCCCAAGCTTATGGGGAGGCCGCCAT | | *Hind* III |
|  | | TaMYB84-SR | | GCTCTAGAAAACATGATGGGGTT | | *Xba* I |
| Transgenic *Arabidopsis* | | TaMYB80-GF  TaMYB80-GR | | GGGGACAAGTTTGTACAAAAAAGCAGGCTTCATGGGGCGCGCGCCGT  GGGGACCACTTTGTACAAGAAAGCTGGGTCTTACATGTAGTCGCTCAC | | attB-site  attB-site |
| RT-PCR | | TaMYB80-RTF | | CAGATGCTCCTCCCTTGG | |  |
|  | | TaMYB80-RTR | | GTGATCCTGGTGTAGTTGC | |  |
|  | | Actin2-F | | GCTCCTCTTAACCCAAAGGC | |  |
|  | | Actin2-R | | CACACCATCACCAGAATCCAGC | |  |
